# Beyond criminal intent: infant-directed violence as a symptom of postpartum psychosis

**DOI:** 10.3389/fpsyt.2026.1975069

**Published:** 2026-10-15

**Authors:** Brooke Laufer

**Affiliations:** Private Practitioner, Evanston, IL, United States

**Keywords:** delusions, forensic, infanticide, postpartum psychosis, risk assessment

## Abstract

Postpartum psychosis is a rare but severe psychiatric emergency in which infant-directed violence, although uncommon, is best understood as a manifestation of acute psychotic illness rather than enduring maternal hostility or conventional criminal intent. The psychotic content of postpartum psychosis is often infant focus, with profound disturbances in reality testing that transform the mother’s perception of her infant, framing the child through delusions and hallucinations as an object of danger, salvation, contamination, or divine significance. In the cases of infanticide or filicide, the violence directed at the child, although objectively tragic are subjectively protective or morally necessary. Drawing on the existing literature on postpartum psychotic phenomenology, this conceptual analysis uses two contrasting clinical cases as illustrative examples—one involving infanticide and one without infant-directed violence—to examine how infant-focused delusions can shape perception and behavior. By contrasting these cases, the article demonstrates how infant-focused delusions directly dictate behavior and explores why the infant becomes the primary object of the mother’s psychotic experience. Finally, the paper discusses the implications of this comparative framework for forensic assessment, criminal responsibility, violence risk evaluation, and clinical intervention.

## Introduction

Postpartum psychosis is a psychiatric emergency associated with an increased risk of suicide (5%) and, more rarely, infanticide (4%), although the vast majority of women with postpartum psychosis never harm their children (1, 2). Unlike many psychotic disorders, postpartum psychosis frequently organizes around the infant, making the mother’s greatest attachment the central object of her psychotic experience. Studies examining the content of psychotic symptoms have identified recurring themes involving the infant, including fears that the baby is harmed or beyond saving, beliefs that the infant is possessed or demonic, convictions that the baby has been replaced or does not belong to the mother, and delusions that ordinary events hold extraordinary significance related to the infant or motherhood (3, 4).

Among women who commit infanticide in the immediate postpartum period, postpartum psychosis has been identified as a significant contributing factor, with some studies estimating that approximately two-thirds of these cases involve psychotic illness (5, 6). The literature suggests that symptoms of postpartum psychosis, such as delusions involving the infant, religious and persecutory delusions, and command hallucinations, may transform the infant into an object of danger, contamination, salvation, or divine significance. Within this altered reality, destructive acts may be experienced by the mother as morally necessary, protective, or salvific despite being objectively tragic and unlawful. This phenomenology differs fundamentally from motives more commonly associated with criminal homicide, such as anger, revenge, financial gain, or sadism.

Women with postpartum psychosis whose delusions specifically center on the infant may represent a different level of clinical risk than women experiencing psychosis without infant-focused delusions or command hallucinations. Yet relatively little attention has been devoted to understanding how the infant becomes the central object of the mother’s psychotic experience and how these symptom patterns may inform forensic assessment.

This article offers a conceptual analysis supported by two unpublished clinical case examples, used as illustrative material to develop and clarify the proposed theoretical framework. The cases examine the phenomenology of infant-centered psychosis in postpartum psychosis and suggest that understanding the symbolic and clinical transformation of the infant within the mother’s altered reality has important implications for violence-risk assessment, criminal-responsibility evaluations, and forensic mental health practice.

## Literature context

Psychosis is characterized by a significant detachment from reality, reflecting disruptions in neuropsychiatric functioning that affect how beliefs arise, persist, and, in some cases, resolve despite clear contradictory evidence. Given the apparent irrationality of delusional beliefs, they have historically been viewed as incomprehensible (7) or fundamentally distinct from ordinary beliefs (8, 9). However, research over recent decades has demonstrated that delusions come from real changes in the brain and how people think, identifiable neuropsychiatric processes and cognitive anomalies (10–12). From a cognitive neuropsychiatric perspective, delusions can be understood as disruptions in the cognitive mechanisms underlying normal belief formation, evaluation, and revision (12, 13). As a result, individuals experiencing psychosis may experience profound alterations in how they perceive and assign meaning to people, relationships, and events, leading to fundamentally distorted beliefs about themselves, others, and the world around them.

In the acute psychosis that follows childbirth, referred to in this article as postpartum psychosis, the clinical presentation exhibits characteristic features that distinguish it from psychotic disorders occurring outside the postpartum period, regardless of whether it is diagnosed as bipolar disorder with psychotic features, major depressive disorder with psychotic features, brief psychotic disorder, or another psychotic illness. Brockington and colleagues found that women with postpartum psychosis demonstrated greater mood lability, distractibility, confusion, impaired sensorium, bizarre delusional content, and memory impairment than patients with psychosis unrelated to pregnancy (14). Similarly, Wisner and colleagues (15) reported that psychosis with onset following childbirth was characterized by marked thought disorganization, delusions of reference, persecutory delusions, and higher rates of homicidal ideation and behavior. Additional reports have described visual, tactile, and olfactory hallucinations, as well as presentations resembling delirium (16). Compared with women experiencing bipolar disorder outside the postpartum period, women with postpartum psychosis were also more likely to experience delusions of control—a sense that an external or overpowering force was directing their thoughts or actions (17). In some cases, these disturbances include command hallucinations instructing the mother to harm herself or her infant, with the commands experienced as authoritative or impossible to resist.

Among the characteristic psychotic themes of postpartum psychosis described in the literature, two are especially prominent: religious or grandiose delusions and infant-centered delusions. Religious delusions may involve beliefs that God has issued commands, that the mother or infant has a special divine mission, or that a sacrificial act is necessary for salvation (3, 4). Infant-centered delusions commonly involve beliefs that the baby is evil, possessed, damaged, replaced, divinely chosen, or must be protected from a fate perceived as worse than death (4, 18, 19).

### The infant as the object of fixation in postpartum psychosis

In the majority of cases of postpartum psychosis, the infant is not merely incidental to the psychosis—the infant becomes central to it. Delusions and hallucinations frequently organize around the baby, with themes of possession, contamination, substitution, divine mission, salvation, or rescue. Chandra and colleagues (18), who examined the prevalence and nature of infant-related delusions among women with postpartum-onset severe mental illness, found that 53% of women with postpartum psychosis experienced delusions involving their infant. These included beliefs that someone would harm or kill the baby, or that the infant would be harmed by the mother’s breast milk. Similarly, Engqvist et al. (20) found that every woman in their qualitative study described delusions that something was fundamentally wrong with the baby or that the infant was ill or dying. Other studies examining the phenomenology of postpartum psychosis have identified recurring themes including fears of harming the baby, beliefs that the mother is unable to care for the infant, delusions that the baby is possessed, and ideas of reference in which ordinary events acquire special significance related to the infant or motherhood (3, 4).

Several hypotheses have been proposed to explain why mothers with postpartum psychosis often become intensely fixated on their infants. One broad explanation is that psychosis tends to organize around a person’s deepest fears, hopes, attachments, and emotionally meaningful relationships (21). In postpartum psychosis, it is therefore unsurprising that the infant—the mother’s primary attachment and greatest emotional investment—frequently becomes the central object of the psychotic experience.

A second explanation emphasizes the unique biology of the postpartum period. The normal maternal drive to protect, monitor, and bond with the infant may become hyperactivated and distorted by severe mental illness. Both the literature and the clinical cases presented in this article suggest that mothers experiencing postpartum psychosis are often highly attentive to their infants and deeply invested in providing exceptional care. Women with postpartum psychosis have been described as conscientious, intensely attached to their infants, frightened of making mistakes, and consumed by a heightened sense of maternal responsibility (14, 22). Rather than reflecting an absence of maternal attachment, the infant may become incorporated into the psychotic process precisely because the attachment is so strong. This interpretation is supported by Gilden et al. (23), who found significantly less impairment in mother–infant bonding among women with postpartum psychosis than among women with severe postpartum depression.

A third perspective draws on psychodynamic theory, which conceptualizes the infant as the focus of unconscious conflict during a profound developmental transition. Motherhood can activate latent fears of inadequacy, guilt, dependency, aggression, and unresolved relational experiences, particularly those rooted in the woman’s own early attachment relationships. From this perspective, an intense preoccupation with the infant may represent an attempt to manage unconscious anxieties through overprotection, excessive responsibility, or symbolic attempts at rescue or control. Richard Lucas argued in The Psychotic Wavelength (2009) that puerperal psychosis is not merely a biological disorder but also a profound psychic crisis reflecting deep maternal anxieties and unconscious fears. He proposed that, although acute psychiatric treatment is essential, long-term recovery may also benefit from psychoanalytic work that helps mothers understand and integrate the psychological meaning of their psychotic experiences. Consistent with this perspective, this author (24) has suggested that, once psychosis has resolved, clinicians may help mothers recover by exploring delusions and hallucinations as symbolic expressions of psychological distress rather than viewing them solely as products of biological dysfunction. Such an approach may facilitate emotional integration, reduce shame, and support long-term recovery.

### Transformation of the infant in the mother’s psychotic experience

One of the defining phenomenological features of postpartum psychosis is the transformation of the infant from an ordinary child into the central object of the mother’s psychotic experience. The infant becomes imbued with extraordinary meaning, serving as the focus of delusions involving danger, salvation, possession, contamination, persecution, or divine purpose. Rather than perceiving the infant as he or she exists in reality, the mother experiences the child through the altered logic of psychosis. Although at times the perception is a positive or neutral one, that the child is a God or savior or the reincarnation of a deceased relative, more frequently the baby becomes a “bad object.”

This transformation shares features with delusional misidentification syndromes (DMS), a group of psychotic phenomena in which the identity of a person is fundamentally altered within the individual’s delusional reality. In postpartum psychosis, the infant ceases to be an ordinary baby and instead acquires a new identity within the mother’s psychotic world. Mothers may develop delusions that the infant has been replaced, is an impostor or “changeling,” or is no longer truly their child (25, 26). Mothers have believed that the baby is evil, possessed by the devil, cursed, threatening, or otherwise dangerous (26, 27). In the 2006 study of the delusional content of mothers experiencing postpartum psychosis, Chandra and colleagues found that the most common psychotic perception was that the infant was a devil or was ill-fated (29 of 56 mothers). Other mothers believed that the infant belonged to someone else, had not yet been delivered, or had been killed by another person.

Similarly, in the present author’s qualitative study (24) of women who had recovered from postpartum psychosis, mothers consistently described profound alterations in how they experienced their infants. Some believed the baby was possessed, evil, or lacked a soul. Others believed the infant was irreparably damaged, already dead, or destined to suffer. Several mothers believed their child had been replaced or was no longer truly their own, while others believed the infant was the target of overwhelming evil or imminent catastrophe. In some cases, mothers believed the child had to die to fulfill a divine purpose, save humanity, or protect the infant from a fate perceived as far worse than death. Across these accounts, the infant was transformed from a loved child into a symbolic figure within an elaborate psychotic narrative.

Importantly, the content of these infant-focused delusions appears to overlap substantially between women who commit infanticide and those who do not. Infant-centered delusions involving divine mission, impending catastrophe, possession, evil, salvation, or protection have been described in both groups (1, 28).

### Infant-directed violence in postpartum psychosis

When violence occurs during postpartum psychosis, it is most commonly directed toward the infant or the mother herself rather than toward others (29). Unlike most forms of interpersonal violence, the violence observed in postpartum psychosis is typically organized around the infant as the central object of the mother’s psychotic reality. Rather than reflecting enduring hostility, anger, or criminal motivation, infant-directed violence emerges within an altered reality in which the mother’s perception of the child has been fundamentally transformed.

As noted, delusional misidentification syndrome (DMS) can transform the perceived identity of a familiar person; importantly, such misidentification has also been associated with hostility and violence toward the misidentified individual (30). In the systematic review by Lewis and colleagues (26), nearly half of the reported postpartum psychosis cases involved some form of infant misidentification. In several cases, mothers refused to care for their infants because of firmly held delusional beliefs. One mother believed so strongly that the infant was not her child that she could no longer bring herself to feed the baby, while another became withdrawn, expressed persecutory ideas, and neglected the infant. As the mother believes that the infant has been replaced, is possessed, is no longer truly her child, or has acquired a threatening identity, her behavior toward the infant becomes organized around these beliefs (26). Chandra and colleagues (18) found that mothers who believed their infant was the devil, ill-fated, or someone else’s baby were more likely to engage in serious abusive behavior and were more likely to “to have significant incidents of shouting at the baby, hitting the baby, or smothering the baby,” (pg. 287) and to be judged unsafe to care for the infant independently.

Importantly, mothers who do not become violent often describe similar psychotic content. In the present author’s qualitative study (24), women recovering from postpartum psychosis who never harmed their infants nevertheless reported intrusive urges or psychotic thoughts of placing the baby in a wood stove, stabbing the infant, driving off a bridge with the infant, or experiencing an overwhelming need to get the baby away from them. These experiences occurred within the context of acute psychosis and were profoundly ego-dystonic once the illness resolved.

From a phenomenological perspective, the infanticidal mother typically does not experience herself as committing murder or acting with criminal intent. Instead, her behavior is organized around the internal logic of the delusional system. Literature on psychosis explains how delusion can create a new moral framework (7, 31). For mothers experiencing psychosis, killing the child may be perceived as morally justified or necessary—to save the child, protect the family, fulfill God’s will, or prevent a far greater catastrophe. The behavior is congruent with the psychotic belief system rather than with societal laws or objective reality. The act may be experienced as rescuing the infant, preventing unbearable suffering, obeying God’s command, protecting the child from evil, or averting an impending catastrophe. Within the psychotic reality, homicide may be perceived as an act of mercy, protection, obedience, or salvation rather than one of malice or revenge. The mother’s actions are therefore experienced as morally, ethically, and even legally justified within her altered reality.

Once the psychosis resolves, this altered moral reality collapses. Women commonly experience profound horror, grief, and an inability to reconcile their actions with their premorbid identity (32). The beliefs that once seemed unquestionably true are recognized as symptoms of severe mental illness, often leaving the mother devastated by what occurred. This phenomenology distinguishes infant-directed violence in postpartum psychosis from filicide motivated by abuse, retaliation, neglect, concealment, or instrumental gain. Rather than reflecting hostility toward the infant or criminal intent, the behavior emerges from a psychotic state in which reality, morality, and judgment have been fundamentally distorted.

## Clinical vignettes

The following cases are previously unpublished composite clinical vignettes derived from women evaluated and treated in both private practice and forensic settings over the past several years. All identifying information has been removed or altered to protect confidentiality, and any verbatim quotations are included with permission. The cases are presented as illustrative clinical material supporting the proposed framework, rather than as empirical evidence establishing a causal relationship between postpartum psychosis and infant-directed violence. Both women developed postpartum psychosis in which the infant became the central focus of the psychotic process. The first case illustrates infant-centered psychosis in which timely recognition and treatment prevented infant-directed violence despite thoughts of harming the infant. The second illustrates a similar psychotic presentation that progressed without effective intervention and culminated in infanticide.

### Case 1. infant-centered postpartum psychosis without infant-directed violence

Case 1 illustrates infant-centered postpartum psychosis in which timely intervention prevented infant-directed violence.

Esther developed postpartum psychosis within weeks of giving birth to her first child. Initially, she experienced severe anxiety, obsessive thoughts, and profound insomnia. She recalled, “I was still crying all the time. I couldn’t sleep. Was she breathing? Was her nose in the bedsheet? Was she getting enough milk?” Although deeply devoted to her daughter, she also experienced frightening intrusive impulses: “The baby’s piercing cry made me want to throw her and my dog across the room.” Dummy **Table 1**

**Table 1:** Comparison of infant-centered psychotic phenomenology in two cases of postpartum psychosis.

Psychotic theme	Case 1 (Esther)	Case 2 (Mary)
Infant’s role in the delusion	Baby chosen by Jesus to announce salvation	Baby targeted by the devil and in need of salvation
Belief about the infant	Baby could walk if released because she possessed miraculous powers	Baby could only be protected by death and taken to heaven
Perception of the world	The end of the world was imminent	Hell had already arrived
Perceived purpose of death	Sacrifice of herself and the baby would save humanity from evil	Death would protect the baby from the devil and allow her to reach heaven
Psychotic experiences	Religious delusions and voices believed to be from God	Religious delusions with command hallucinations directing harm to the child and herself

As her illness progressed, her anxiety evolved into paranoia and mania. She became convinced that “a gentleman on his cellphone in a black SUV was reporting my whereabouts to the FBI.” She questioned whether she had somehow become a national security threat: “Did the FBI think I did something to that airplane? Was I suddenly a national terror threat? My mind was convinced something much bigger than me was happening.” Esther described the onset of psychosis as rapid, with her beliefs becoming increasingly bizarre over only a few days.

The psychosis quickly organized around her infant through grandiose religious delusions. Esther became convinced that the end of the world was imminent and that she and her daughter had been chosen by God. “The end of the world was coming. I was chosen by Jesus himself to have this beautiful baby girl and announce to the world that salvation was near.” She began approaching strangers, shaking their hands and telling them to smile because Jesus loved them. She believed her daughter was a prophet and possessed miraculous abilities. While introducing the baby to her brother, she heard what she believed was the voice of God saying, “My nine-week-old baby could walk if I just let her go.” The voice reassured her that “it was okay if she died, or if I died, because we would be saving the world from all evil.”

Her religious beliefs, which had previously been a source of comfort, became incorporated into the psychosis. “My faith—something I’d always relied on to keep me grounded—was now working against me.” She told her brother that the world was ending, that he should empty his house of unnecessary possessions, and insisted, “Look, my baby can walk on water. She can walk.” At times she spoke very little, stating only, “I am a prophet … Saint Jesus.” She increasingly believed she and her baby needed to die in order to save the world.

The paranoia intensified alongside the infant-centered delusions. Esther reported checking behind doors and shower curtains because she believed the Secret Service was following her. “I prayed that I would be saved, and my life would be spared.” Her family recognized that something was seriously wrong and urged her to contact her obstetrician. Her OB/GYN immediately instructed her to present to the emergency department, telling her, “This is an emergency.”

Despite disclosing her paranoia and bizarre beliefs during her emergency psychiatric evaluation, Esther recalled being told to “snap out of it” and to ignore her thoughts. She was discharged with a single dose of Xanax. She subsequently saw both her therapist and psychiatrist, but although medication was prescribed, her psychosis continued to worsen. “In my head I was still fighting for my life—in a world full of undercover agents looking to kill me.”

Over the following weeks, Esther remained consumed by persecutory and religious delusions. She interpreted internet security alerts about password breaches as evidence of a government conspiracy to imprison and eventually execute her. She changed the family’s Wi-Fi password, unplugged electronic devices so the government could not monitor her conversations, and became convinced that the media had surrounded her home. At one point she locked herself in the bathroom and called the White House Whistleblower Hotline because she believed she possessed critical information about an airplane conspiracy.

Although her obstetrician had correctly recognized postpartum psychosis, Esther reported that few others around her—including mental health professionals—understood the severity of her condition. Eventually, a family member who had attended a conference hosted by Postpartum Support International recommended a specialized maternal mental health program. Esther was admitted to a residential mother-baby psychiatric unit where she received intensive treatment, medication management, and family involvement until her psychotic symptoms resolved. She made a full recovery, later had another child without recurrence of psychosis, and continues to function well.

### Case 2. infant-centered postpartum psychosis with infanticide

Case 2 illustrates infant-centered postpartum psychosis in which psychotic symptoms progressed without effective intervention, culminating in infanticide.

Mary lived with her father and sister and was separated from her daughter’s father, although he remained actively involved and visited regularly. Although the pregnancy had been unplanned, Mary described motherhood as something she had always wanted. She was intensely devoted to her daughter and focused on making every aspect of pregnancy, birth, and infancy as perfect as possible. She later explained that she wanted to give her daughter “the childhood I never had.” She prepared all of her daughter’s food from scratch, read and sang to her daily, remained highly protective of her wellbeing, and consistently breastfed.

When her daughter was approximately 4 months old, Mary began experiencing severe anxiety and an unshakable conviction that “something bad was going to happen.” Initially, the psychosis emerged through persecutory beliefs. She became preoccupied with drones, convinced they were surveilling her neighborhood. After photographing what she believed was a drone, she increasingly believed that drones were watching people and might “release a gas that would kill us.” She occasionally shared these concerns with her sisters, explaining that they did not understand because “they didn’t watch the news.” She spent hours watching YouTube videos about drones and international conflicts, particularly news coverage of Syria, which left her increasingly fearful and “on edge.”

Throughout this period, Mary’s attachment to her daughter remained intense. As her psychosis progressed, her longstanding vigilance regarding her daughter’s safety became increasingly distorted by delusional beliefs.

In the days preceding the offense, Mary’s thinking deteriorated rapidly. She became convinced that she was being watched through her phone and television and avoided speaking openly because she believed “they were being watched.” She described feeling constantly surveilled and “spied on.” She reported seeing “hazy smokiness” coming from the vents in the home and became preoccupied with cleaning them. She scanned constantly for dangers that might threaten her daughter. At one point she instructed her sister to “write down what you say because we’re being watched.” She covered the television in her bedroom with a blanket and developed fears that bombs might soon be dropped nearby.

The night before the offense, the child’s father visited the home. Mary reported seeing what appeared to be a triangle on his forehead, which she interpreted as being “related to the devil.” After the parents read a children’s book together with their daughter, Mary became convinced that the book itself was “a doorway for spirits.” She asked him to leave the home and barricaded her bedroom door with a dresser.

By the following morning, the psychosis had become profoundly religious and infant-centered. Mary awoke believing that she “was in hell.” Christmas lights outside appeared to her as flames, confirming that belief. She reported feeling “possessed” and that she “didn’t have control over” herself. Clutching her daughter in an effort to protect her, she repeatedly asked why they were in hell.

Mary became convinced that members of her family were “demons” and that the devil was coming to take her daughter. She also developed bizarre persecutory beliefs that men were “raping women, impregnating women, making women have multiples.” She then heard a clear voice telling her that the devil was going to eat the baby but that the baby could still go to heaven. As the voices intensified, she began shaking uncontrollably.

Mary prayed and repeatedly asked God what she should do. She believed God instructed her to “grab a blanket.” Her memory then became fragmented. She recalled hearing commands directing her to end both her daughter’s life and her own. The voice instructed her to “take her breath” by suffocating her daughter with a pillow, which she did. Mary later explained that she had “no control of my thoughts or my body” and felt as though she was “watching my body do things I couldn’t stop.”

She also heard that the “book of commandments was not real” and that she and the child needed to “go to the water” to escape hell. She filled the bathtub with cold water because she believed it would allow them to “get to heaven.” The voices simultaneously instructed her to end her own life. She retrieved a knife from the kitchen, attempted to cut her wrists and stab her abdomen, and also attempted to drown herself.

When emergency responders arrived, Mary was naked, shaking, praying, and describing herself as “trapped.” She was transported to the hospital, where she was treated with antipsychotic medication. During her hospitalization, she continued expressing bizarre beliefs, telling staff that “we’re brothers and sisters” and describing herself as trapped inside a “program” or “system.”

Within approximately 3 weeks of antipsychotic medication, Mary’s psychotic symptoms resolved. She later described the person she had been during the preceding weeks as someone who “wasn’t me.” Following her recovery from psychosis, she was charged with infanticide and remains involved in criminal proceedings.

## Discussion: comparison of te two cases

Although the content of the delusions differed, both psychotic episodes organized around the infant as the primary object of meaning. In each case, the mother’s most valued attachment became incorporated into an elaborate delusional system involving salvation, supernatural forces, and protection from catastrophic harm.

For both mothers, this was their first child—a wanted child to whom they were deeply devoted. Their early maternal responses were characterized by attentiveness, protectiveness, and a strong desire to be good mothers. This was especially evident in Mary, whose identity became closely tied to providing her daughter with the childhood she had not experienced herself. In both cases, an underlying vulnerability to anxiety appeared to intensify following childbirth, progressing from overwhelming anxiety and hypervigilance into increasingly disorganized thinking, mood elevation, and ultimately psychosis.

In both cases, the infant ceased to be experienced as an ordinary child and instead acquired extraordinary psychological meaning: a prophet, a savior, a target of evil, or a gateway to heaven. The mothers’ fundamental instinct to protect their children was not absent; rather, it was transformed by psychosis into behaviors experienced as necessary, urgent, and morally justified within their altered reality. The protective maternal system became distorted by delusion, leading to actions aimed at achieving what the mother believed was the child’s ultimate safety or salvation.

Both mothers experienced a profound loss of insight and significant impairment in reality testing. In each case, the transition from feeling overwhelmed, exhausted, or disconnected to experiencing a fully developed psychotic state appeared to occur rapidly, described by both women as a kind of “switch” into an altered reality. Within this state, their experiences became organized around elaborate delusional narratives infused with paranoia, perceived threats, and beliefs that powerful external forces were targeting them. Both women believed they were being watched and monitored: Mary through her television, phone, and perceived surveillance systems, and Esther through her belief that the Secret Service and government agents were following her.

Within these delusional realities, the mothers themselves also became central targets of danger. For Mary, command hallucinations instructed her that she needed to end both her child’s life and her own. During the episode, she attempted to harm herself as well as her daughter. For Esther, the belief that she and her child needed to die was framed within a religious delusion that their sacrifice would save the world from evil.

Although Esther did not act on these beliefs, her psychosis contained elements that could have resulted in serious harm. She believed that allowing her infant to “walk” or releasing her child from her protection was a necessary act within her delusional framework. Without intervention and treatment, the progression of her psychosis could have potentially escalated to infant-directed violence. These cases illustrate that the presence or absence of violence is not necessarily a reflection of maternal attachment or intent but may instead depend on the trajectory of the psychotic process, the content of the delusions, and the timing of intervention.

### Application of the theoretical framework to the clinical vignettes

The two clinical vignettes illustrate different theoretical pathways through which the infant may become incorporated into postpartum psychosis. Esther’s case most clearly illustrates the first hypothesis, that psychosis may organize around a person’s deepest fears, attachments, and emotionally meaningful relationships. Her infant, as her primary attachment and greatest emotional investment, became central to grandiose and religious delusions concerning salvation, divine selection, and the end of the world.

Mary’s case is more consistent with the second and third hypotheses. The second hypothesis proposes that the normal maternal drive to protect, monitor, and care for the infant may become hyperactivated and distorted by psychosis. Mary’s intense vigilance regarding her daughter’s safety progressively became organized around persecutory fears that the child was being threatened by demons, surveillance, and other dangers. The third, psychodynamic hypothesis proposes that the infant may become the focus of unconscious conflicts involving maternal adequacy, responsibility, vulnerability, and unresolved relational experiences. Mary’s determination to give her daughter “the childhood I never had,” together with her profound sense of maternal responsibility, provides a possible context for understanding how these concerns became incorporated into her psychotic experience. Thus, while Esther’s psychosis illustrates the role of attachment and emotional salience, Mary’s case illustrates how an intensified maternal protective drive and deeper psychological conflicts may become transformed within postpartum psychosis.

### The role of intervention

The primary difference between these two cases was not the presence or absence of infant-centered psychosis, but the timing and availability of intervention. Esther had a supportive network that included her husband and parents, who recognized that something was seriously wrong and encouraged her to contact her physician. Her obstetrician had sufficient awareness of postpartum psychosis to recognize that Esther’s symptoms represented a psychiatric emergency and instructed her to seek immediate evaluation in the emergency department.

Esther’s stabilization required multiple treatment attempts, as initial medications did not immediately resolve the psychosis. However, she ultimately benefited from an informed support network that connected her with specialized maternal mental health care. She was admitted to a mother-baby psychiatric residential program, where she continued to be surrounded by family while receiving medication management and therapeutic support. Intervention occurred at a critical point in the progression of her illness—after her delusions had transformed her perception of her infant and as her beliefs about danger and salvation were intensifying, but before those beliefs resulted in harm.

Mary’s course differed not because her symptoms were less severe, but because the pathway to intervention was not activated. Although Mary communicated aspects of her paranoia to her sisters and expressed that something was wrong, there was uncertainty about how to respond and limited recognition of the severity of her condition. She had successfully mothered her daughter for many months, and her family had not observed obvious psychiatric deterioration before the acute escalation of symptoms. When her psychosis intensified, it progressed rapidly.

Unlike Esther, Mary was also more isolated. Although her father and sisters were involved in her life, she did not have a partner in the home, and she and her daughter often spent significant time alone in their room. There was no intervention to prompt an emergency psychiatric evaluation before the crisis occurred. Treatment ultimately came after the offense, and it was successful in resolving her psychosis, but only after devastating consequences had occurred.

### Implications for assessment, treatment, recovery, and risk reduction

Assessing the content of psychotic symptoms may improve violence risk assessment more than diagnosis alone. Rather than viewing postpartum psychosis as a uniform risk factor for infanticide, clinicians should evaluate the dynamic meaning and phenomenological content of psychotic symptoms, particularly the mother’s representation of the infant. Delusions in which the infant has become transformed into a persecutory, demonic, contaminated, possessed, or otherwise altered object may be more informative for risk assessment than the diagnosis of postpartum psychosis alone. Recognizing these recurring themes can improve clinical decision-making, guide intervention, and inform recovery-oriented treatment.

Improved screening for postpartum psychosis is also essential. Although the Edinburgh Postnatal Depression Scale (EPDS) has become routine in many obstetric practices, it was developed to screen for postpartum depression and includes only limited assessment of psychotic symptoms. More recently, researchers have developed instruments specifically designed to identify postpartum psychotic experiences. One such measure is the Postpartum Psychotic Experiences Scale (PPES), a 15-item self-report instrument introduced in 2022 to assess early or mild psychotic symptoms during the postpartum period (33). The PPES measures experiences such as unusual thought content, perceptual disturbances, and disorganized or fragmented thinking and communication, with the goal of improving early identification of women who may be at increased risk for postpartum psychosis while also providing a standardized measure for research. Despite the promise of structured screening instruments, the most comprehensive method of assessment remains a detailed clinical interview conducted by a clinician with expertise in postpartum psychosis, allowing for careful evaluation of symptom content, insight, functional impairment, and the phenomenology of the mother’s psychotic experience.

Clinicians should routinely assess for psychotic symptoms, including persecutory beliefs, thought disturbance, hallucinations, and changes in the perceived identity of the infant or other family members. Useful screening questions include: Do you feel that your thoughts are being heard or known by other people? Do you feel that another person or an outside force is controlling your thoughts or actions? Have you heard voices or sounds that others do not seem to hear? Have you seen visions or things that others do not seem to see? Have any people, including your baby, seemed different or as though they have changed identity? Assessment can be challenging because mothers experiencing postpartum psychosis are often guarded, suspicious, or reluctant to disclose psychotic experiences due to fear, confusion, or impaired insight. A gradual, empathic interview facilitates sensitive exploration of these symptoms while allowing for a more accurate assessment of the underlying psychopathology.

Assessment should also extend beyond the mother to include family members whenever possible. Family members frequently observe behavioral changes before the mother recognizes that she is becoming ill. Asking questions such as, Have you noticed sudden changes in her sleep, behavior, personality, or beliefs? can provide valuable collateral information. Families should also be educated about the early warning signs of postpartum psychosis—particularly rapid sleep loss, increasing paranoia, religious or grandiose thinking, confusion, and infant-focused delusions—so they can seek emergency evaluation promptly. In many cases, collateral information from family members may be more reliable than the mother’s self-report during an acute psychotic episode.

Delusional content may also have implications for recovery. Rather than viewing delusions solely as meaningless products of psychosis, clinicians may benefit from attending to the subjective meaning patients attribute to them. Ritunnano et al. (34), in a systematic review and qualitative evidence synthesis, found that patients’ subjective experiences and meanings of delusions are important dimensions of psychosis that may be overlooked when delusions are approached primarily as symptoms to be eliminated. Similarly, Laufer (24) found that the content of postpartum psychosis often reflects patients’ fears, conflicts, and concerns, suggesting that psychotic experiences may carry personal meaning. This perspective may be particularly relevant when the infant becomes incorporated into the mother’s delusional system: understanding what the infant represents within that system may provide insight into the risk of infant-directed violence while also creating opportunities for therapeutic engagement and recovery.

Finally, greater recognition of postpartum psychosis as a distinct clinical syndrome would improve assessment, treatment, and prevention. Formal inclusion of postpartum psychosis as its own diagnostic entity in future editions of the DSM would likely stimulate research, improve diagnostic consistency, increase clinician education, and promote the development of evidence-based screening and treatment protocols. Such recognition could ultimately improve outcomes for mothers and infants by facilitating earlier identification and intervention.

### Implications for forensic assessment

Understanding infant-directed violence as an expression of acute psychotic illness has important implications for forensic evaluation. Rather than conceptualizing these cases within the framework of conventional criminal violence, evaluators should examine the phenomenology of the psychosis and the extent to which the mother’s behavior was organized by delusions, hallucinations, and profound impairment in reality testing. Particular attention should be paid to the content of the psychotic symptoms, including infant-focused delusions, command hallucinations, religious or grandiose beliefs, persecutory ideation, and alterations in the perceived identity of the infant. These symptoms provide important information regarding the mother’s mental state at the time of the offense and may be more informative than diagnosis alone.

A phenomenological approach also helps distinguish postpartum psychosis from other forms of maternal filicide motivated by abuse, retaliation, neglect, concealment of pregnancy, or instrumental gain. In postpartum psychosis, the mother’s behavior typically arises from an internally coherent delusional reality in which the infant has acquired extraordinary significance and the act is experienced as necessary, protective, merciful, or divinely commanded. The behavior is therefore qualitatively different from violence motivated by anger, revenge, financial benefit, or a desire to rid oneself of parental responsibilities.

The findings presented in this article further suggest that forensic evaluations should include careful reconstruction of the evolution of the psychosis rather than focusing solely on the homicidal act itself. The onset of insomnia, rapid mood changes, paranoia, religious preoccupation, cognitive disorganization, infant-focused delusions, and command hallucinations often unfolds over days or weeks before the offense and may provide crucial evidence of an emerging psychotic disorder. Collateral information from family members, obstetric providers, emergency department records, therapists, and other treating clinicians can therefore be essential in accurately reconstructing the mother’s mental state at the time of the alleged offense.

Finally, recognizing infant-directed violence as a manifestation of postpartum psychosis has implications for opinions regarding criminal responsibility, violence risk, and disposition. Although legal standards for insanity vary across jurisdictions, forensic evaluators should distinguish psychotically driven violence from criminal acts arising from intact reality testing. Likewise, violence risk assessments should consider that the risk associated with postpartum psychosis is largely state-dependent. For many women, the risk of infant-directed violence resolves with successful treatment of the psychosis and does not necessarily reflect an enduring propensity for violence. Understanding this distinction can improve forensic decision-making while promoting clinically informed, evidence-based opinions regarding culpability, treatment needs, and future risk.

## Conclusion

Active exploration of the content and phenomenology of psychotic symptoms in postpartum psychosis is essential for reducing the risk of infant-directed violence, preserving the mother–infant relationship, and improving clinical outcomes. Careful assessment of infant-focused delusions, hallucinations, and alterations in the mother’s perception of her infant may also reveal that these symptoms are more common in postpartum psychosis than has previously been recognized. Early identification and treatment remain the most effective means of preventing tragedy.

From a forensic perspective, the central question is therefore not simply whether a mother experienced postpartum psychosis, but how the illness transformed her perception of herself, her infant, and the moral reality within which she acted. Understanding the phenomenology of postpartum psychosis—and particularly the transformation of the infant within the mother’s psychotic world—provides a more accurate framework for clinical assessment, violence risk evaluation, criminal responsibility, and recovery than diagnosis alone.

### Limitations and ethical considerations

This paper is based on two unpublished clinical vignettes and is intended to provide clinical and theoretical illustration rather than empirical evidence of a causal relationship between the content of postpartum psychosis and infant-directed violence. The small number of cases limits the generalizability of the observations; however, the detailed clinical material permits qualitative consideration of how the infant may become incorporated into the mother’s delusional system and how this may be relevant to risk. The cases are presented as anonymized clinical material, with potentially identifying information removed or modified. Verbatim patient quotations are included with permission. No institutional support or authorization was required for the use of this clinical material.
